# INEQUALITIES IN POLYPHARMACY IN ADULTS AGED 65+ IN ENGLAND

**DOI:** 10.1093/geroni/igad104.1795

**Published:** 2023-12-21

**Authors:** Anum Iqbal, Barbara Hanratty, Fiona Matthews, David Sinclair, Connor Richardson, Adam Todd

**Affiliations:** Newcastle University, Newcastle upon Tyne, England, United Kingdom; Newcastle University, Newcastle upon Tyne, England, United Kingdom; Newcastle University, Newcastle upon Tyne, England, United Kingdom; Newcastle University, Newcastle upon Tyne, England, United Kingdom; Population Health Sciences Institute, Newcastle Upon Tyne, England, United Kingdom; School of Pharmacy, Newcastle Upon Tyne, England, United Kingdom

## Abstract

Understanding the role of socio-economic factors in the number of medications an individual is prescribed, can help healthcare practitioners target medication reviews and safer prescribing, improving quality of life. We aimed to understand the association between socio-economic factors and polypharmacy - defined as the use of five or more medications at any given time. Data was used from the Cognitive Function and Ageing Study (CFAS), focusing on adults aged 65 years and over. Data from CFAS I and CFAS II, was used to understand the relationship between education (a marker of socio-economic status) and polypharmacy. Individuals were categorised on the number of years in full time education, and baseline cohorts (wave 1) from each study were compared, to identify the longitudinal effect. Logistic regression was used to assess the association between polypharmacy and education. Increased number of years of full-time education was associated with a decreased likelihood in polypharmacy, in CFAS II - those with <10 years of full-time education showed higher odds of polypharmacy, when compared to individuals with 10-11 years of full-time education, OR 1.61 (95 % CI 1.44-1.80). However, similar odds were displayed across each education category and polypharmacy in CFAS I. Results have showcased growing inequalities in healthcare, particularly through the influence of education on polypharmacy. Socio-economic factors could be used to trigger for medication related review services.

